# Review of time-resolved non-contact electrostatic force microscopy techniques with applications to ionic transport measurements

**DOI:** 10.3762/bjnano.10.62

**Published:** 2019-03-01

**Authors:** Aaron Mascaro, Yoichi Miyahara, Tyler Enright, Omur E Dagdeviren, Peter Grütter

**Affiliations:** 1 Department of Physics, McGill University, 3600 rue University, Montreal, Québec H3A2T8, Canada

**Keywords:** atomic force microscopy, electrostatic force microscopy, ionic transport, lithium ion batteries, nanotechnology

## Abstract

Recently, there have been a number of variations of electrostatic force microscopy (EFM) that allow for the measurement of time-varying forces arising from phenomena such as ion transport in battery materials or charge separation in photovoltaic systems. These forces reveal information about dynamic processes happening over nanometer length scales due to the nanometer-sized probe tips used in atomic force microscopy. Here, we review in detail several time-resolved EFM techniques based on non-contact atomic force microscopy, elaborating on their specific limitations and challenges. We also introduce a new experimental technique that can resolve time-varying signals well below the oscillation period of the cantilever and compare and contrast it with those previously established.

## Introduction

Since the inception of the atomic force microscope (AFM) a variety of techniques have been developed aimed at measuring local electronic and ionic properties on a wide range of samples. By carefully controlling the electric field between the tip and sample many properties can be measured with high spatial resolution including static properties such as local contact potential difference (which can be used to extract the local work function) [1] and local piezoelectric response [2], and dynamic properties such as the charging and decay times of photoexcited carriers [3–6], and local activation energies for ionic transport [7–8]. These measurements play a crucial role in understanding local charge dynamics and composition of numerous materials with applications across many fields including energy generation and storage. Capturing time-resolved dynamic processes at ever-decreasing time and length scales has become of increased interest in recent years due to the importance of understanding transport properties of real-world, often heterogeneous materials relevant for energy generation and storage. A number of AFM techniques have been developed to study relevant materials including time-resolved EFM to measure photoexcited charge accumulation and charge transfer [6,9–11], time-domain EFM to measure ionic transport [7,12], time-resolved electrochemical strain microscopy (ESM) to measure ionic transport [8,13], various time-resolved Kelvin probe force microscopy (KPFM) techniques that utilize either optical pump-probe or advanced signal processing to measure time-resolved surface potentials [14–18], and other techniques that exploit non-linear signal mixing or heterodyning to extract the time evolution of the tip–sample interaction [19–20]. All of these techniques share a common goal of furthering the understanding of charge generation and transport processes to develop a clear picture of the underlying mechanisms that govern them. This requires an extensive toolbox of experimental techniques of which EFM-based ones will most certainly play an essential role.

In this review we explore in detail several techniques that allow for time-resolved electrostatic force measurements to probe ionic transport. More specifically, these techniques are able to capture time-varying changes in the tip–sample coupling due to the movement of mobile ions within the sample in the sample volume directly underneath the probe tip. The ionic motion is initiated by an electric potential applied across the sample; the movement of mobile ions leads to a change in the tip–sample capacitance and, thus, to a change in the electrostatic force acting on the cantilever probe tip. The electrostatic tip–sample force is proportional to the capacitance gradient ∂*C*/∂*z* times the square of the applied potential *V*(*t*)^2^, i.e.,


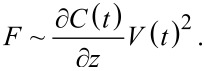


In ionic transport measurements it is the time-dependence of the capacitance *C*(*t*) that is to be measured; however, this is not usually a known quantity. In order to validate a technique for suitability in measuring this quantitatively, a known time-varying voltage with the same functional time-dependence that mimics the expected *C*(*t*) can be used instead. This allows for a quantitative assessment of the extracted parameters and is the method used to validate each technique discussed herein.

Since there is a multitude of similar techniques, each with their own clever implementations and analyses, we have restricted our review to exclude all pump–probe and KPFM techniques as these are generally unsuitable for probing ionic transport. For a recent review of all KPFM and related techniques, we refer the reader to [21].

We begin by describing the direct time-domain method and its limitations, we then introduce a new technique we refer to as voltage-pulse averaging EFM, and then continue to explain and examine three other techniques with applications to ionic transport measurements, specifically fast free time-resolved EFM [22], phase-kick EFM [23], and intermodulation spectroscopy [20]. Table 1 lists these techniques along with their respective time resolutions (smallest value demonstrated), limitations, and strengths.

**Table 1:** Overview of the five techniques explored in this review. Time resolution is the smallest demonstrated value.

technique	time resolution	limitations	strengths

direct time-domain	above 2 μs	– resonance frequency and detection bandwidth limit time resolution	– simple implementation
voltage-pulse averaging EFM	ca. 200 ns	– significant averaging time	– simple implementation
		– difficulty in extracting stretched exponential	– time resolution not limited by detection bandwidth
		– functional form of *C*(*t*) must be known	
fast free time-resolved EFM [22]	ca. 10 ns	– slowly varying relationship between τ and extracted signal *t*_fp_ for sub-cycle time constants	– excellent spatial resolution
		– difficulty in extracting stretched exponential for small time constants	– fast imaging times with simultaneous acquisition and analysis
phase-kick EFM [23]	ca. 35 ns	– requires precisely phase-locked excitation signals	– strong signal-to-noise ratio due to averaging
		– tip–sample force gradient must be approximately constant over oscillation cycle	– time resolution not limited by detection bandwidth
intermodulation spectroscopy [20]	ca. 30 ns	–high Q-factor cantilevers result in lower signal-to-noise ratio	– time resolution theoretically only limited by measurement time
		– a time dependent capacitance will likely lead to a complicated analytic representation needed to extract the time-evolution of the system	– full time-evolution can be captured using only a single measurement

## Review

### Direct time-domain EFM

#### Background

Direct time-domain EFM measurements are the most straightforward methods of measuring time-varying interactions. In the commonly used frequency-modulated AFM configuration, the resonance frequency of an oscillating cantilever is measured while the probe tip interacts with a surface [24]. The interactions are purely electrostatic – in other words, the tip and sample form a capacitor. The oscillation of the cantilever can therefore be modulated by the electric field between the tip and sample, which may vary with time. The first use of an AFM to measure the time evolution of sample charge carriers was reported by Schönenberger and Alvarado [25]. They first applied a voltage pulse between the tip and sample to inject charge into the sample. They subsequently measured the (ac) electrostatic force as a function of time using a lock-in amplifier where the observed force decayed over several seconds.

In the case of photovoltaic samples, simply shining light on them photoexcites charge carriers, which can result in charge build-up in the sample at the location of the AFM tip if an appropriate voltage is applied across the tip–sample gap. Measuring the resonance frequency shift as a function of time after the light is turned on/off then allows for the charging/discharging time to be directly acquired, revealing information about charge generation and transport in the sample. This was first performed by Krauss et al. who observed charging of photoexcited CdSe nanocrystals by direct frequency shift measurements after illumination [26].

**Figure 1:**
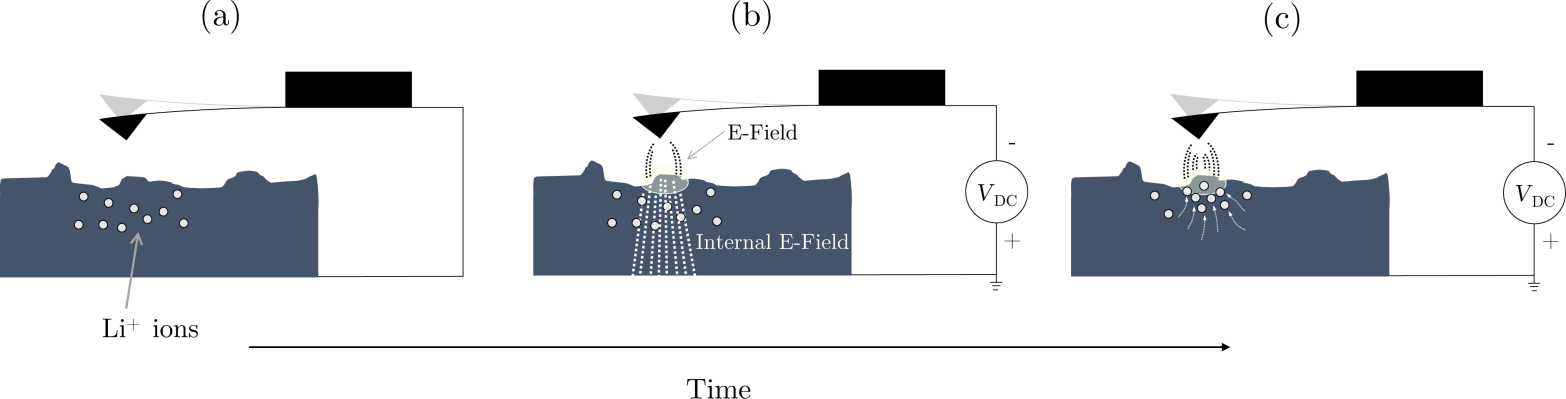
Illustration of ionic transport measurements in the time domain. (a) A conducting AFM tip is brought close (typically 1–20 nm) to the surface of a sample containing mobile ions (Li^+^ in this case). (b) A step potential (*V*_dc_) is applied between the tip and back electrode, creating an electric field that extends through the sample. (c) The mobile ions move towards the tip (in the case of a negative tip bias and grounded back electrode), shielding the internal electric field.

The concept outlined above can be applied to measure ionic transport in ionic conducting materials as well. To probe ionic transport a step potential is applied between the AFM tip and a conducting back electrode, creating an electric field across the tip–sample gap and through the sample, illustrated in Figure 1b. The mobile ions inside the sample move in response to this field over time, resulting in a change in the field (and field gradient) at the tip as illustrated in Figure 1c. This changing electric field as a result of screening by the mobile ions leads to a shift of the cantilever resonance frequency as a function of time, which can be directly measured, typically by using a phase-locked loop (PLL). This was first performed by Bennewitz et al. to measure the mobility of F^−^ vacancies in a CaF_2_ crystal [27]. Schirmeisen et al. later improved the technique by performing the measurements at various temperatures to extract the activation energy for ionic transport in Li^+^ conducting glasses [7]. To further expand the power of the technique, Mascaro et al. developed a real-time averaging system used in conjunction with a fast (high-bandwidth) PLL to improve the time resolution [12]. This enabled ionic transport measurements to be performed on lithium iron phosphate (LiFePO_4_), a relevant lithium-ion battery cathode material. In this configuration the time resolution (and thus the fastest ionic conductor that can be measured) is limited by the time response of the PLL, which depends on many parameters including the free resonance frequency of the cantilever as well as the various PLL settings.

#### Limitation: direct frequency detection

A critically damped second-order PLL (i.e., optimized settings) has an exponentially decaying time response to abrupt changes in the frequency being tracked (the center frequency, *f*_0_) [28]. The response time-constant of the phase detector is determined directly by the center frequency: τ_PD_ = 1/*f*_0_. Thus, the theoretical minimum response time to achieve more than 95% tracking is three cycles. This is difficult to realize in practice as it neglects amplification/filtering before and after the phase detector and other non-ideal effects such as jitter and noise. The overall response time of the system (τ_PLL_, inversely proportional to the overall bandwidth) serves as a more practical metric as it takes all contributions into account. This can either be measured by stepping the frequency of a known signal and measuring the response time or in the case of some digital PLLs by a built-in function that models the response [29].

In general, ionic transport in solid ionic conductors follows a stretched-exponential time response to applied electric fields:

[1]
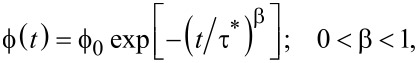


where 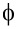 represents the internal electric field, 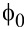 is the initial field strength, β is the stretching factor, and τ* is the collective (or overall) time constant for the response [30]. Note that this stretched exponential behaviour is due to the correlated nature of ion transport, which depends on the atomic and electronic structure of the material, and not necessarily due to a distribution of relaxation times [31]. Nonetheless, this complicates time-domain measurements of ionic transport as the functional form of the relaxation must be fully captured in order to reliably extract the relevant parameters, namely τ* and β. With slow ionic relaxation times (longer than milliseconds) and typical operating (scanning) parameters (bandwidth of ca. 100 Hz) the PLL response will not affect the extracted values obtained from directly fitting the data. However, as the relaxation time approaches the response time of the PLL, the output signal will become a convolution of the PLL response function and the ionic relaxation. This makes any quantification of the transport properties challenging.

**Figure 2:**
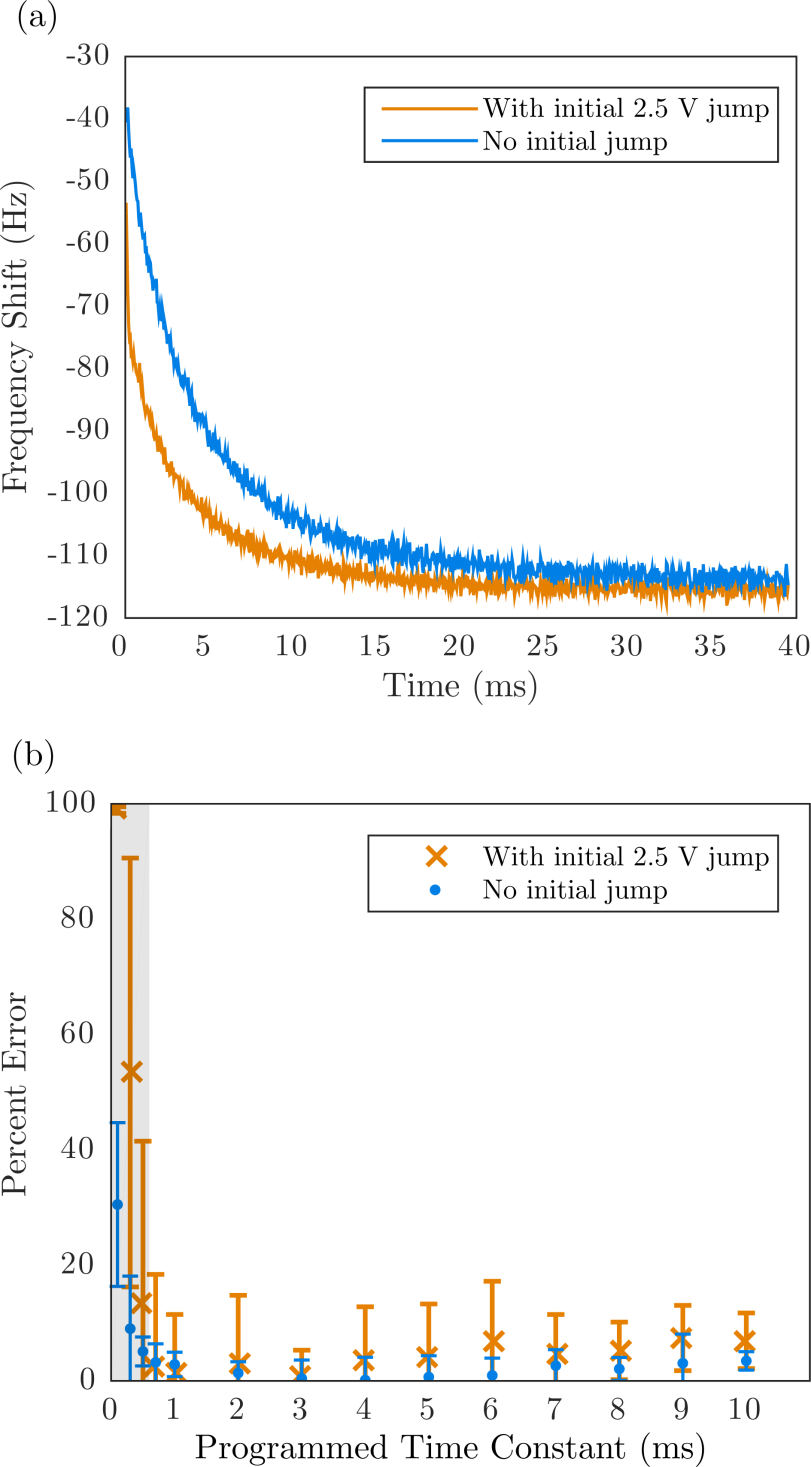
(a) AFM frequency shift response to stretched-exponential voltage pulses (from 0 to 5 V) with and without an intial 2.5 V jump. (b) Percent error of fitted relaxation time constant (τ*) as a function of relaxation time constant of applied voltage pulse for fixed PLL response time (τ_PLL_). Shaded region shows where τ* *<* τ_PLL_.

To investigate the effect of τ_PLL_ on the ability to extract parameters from measured signals, a digitially synthesized voltage waveform varying in time as a stretched exponential (Equation 1, β = 0.7) was applied between a Pt-coated AFM tip and a gold substrate (separated by about 20 nm) under high vacuum (ca. 10^−6^ mbar, Jeol JSPM-5200) to simulate ionic transport in the sample with a known decay time constant. Note that a separation ≥1 nm is necessary in general to ensure that no charge is injected into the sample. In this case, the electric field follows the applied voltage instantaneously on the relevant time scales. For each programmed time constant (from 0.1 to 10 ms), the voltage was varied from 0 V initially to 5 V; the measured response is shown in Figure 2a where the blue curve is the result of the smoothly varying stretched-exponential applied voltage. The orange curve is the result of applying an initial instantaneous jump from 0 to 2.5 V followed by a stretched-exponential increase to 5 V. This is intended to mimic experimental conditions as the step voltage applied causes an initial jump in the resonance frequency (due to the stepped electric field between the tip and sample before the ions respond) followed by the slow sample relaxation (as the ions move to shield the initial electric field). Since the actual time constant is given by the synthesized voltage waveform, the percent error can be directly calculated from the fit results. Note that since the frequency shift is quadratic in voltage and it is the voltage being changed here, we must first take the square root of the data before fitting. The results are shown in Figure 2b where the shaded area is the region for which τ* *<* τ_PLL_ ≈ 600 μs. To replicate measurement conditions, 100 waveforms were applied as a pulse train (50% duty cycle) and the response signals were averaged together using our realtime averaging system to reduce noise (described in [12]). In both cases (with and without the initial jump) we are able to accurately extract the relaxation time constant; the initial jump only leads to a higher statistical uncertainty, which is due to the slow initial response of the PLL relative to the fast jump from 0 to 2.5 V. This becomes especially apparent for τ* ≈ τ_PLL_. The stretching factor displays exactly the same behaviour (not shown).

Clearly the practical limitation for high-fidelity measurements is determined by τ_PLL_ as the percent error increases drastically for τ* *<* τ_PLL_. Simply increasing the PLL bandwidth will decrease τ_PLL_ although this will result in higher noise due to the less aggressive filtering. By taking a larger number of averages, the same signal-to-noise ratio can be achieved, however the cantilever resonance frequency will ultimately determine the minimum τ_PLL_. Empirically we have found that the minimum PLL response time achievable is τ_PLLmin_ ≈ 10 × 1/*f*_0_. The highest resonance frequency cantilevers currently commercially available have frequencies of *f*_0_≈ 5 MHz, thus the realistic minimum measurable relaxation time of this technique is ca. 2 μs.

### Voltage-pulse averaging EFM

#### Motivation

Improving the time resolution beyond the limitations of direct time-domain measurements is possible in several ways using careful instrumentation and signal analysis. The basic concept is to detect (using a slow detector) the change in average response of the sample due to a change in the frequency (or, e.g., repetition rate or delay time) of an excitation signal. This is also the basis for pump–probe spectroscopy, which is routinely employed to measure ultrafast dynamics of condensed matter systems using a variety of pulsed light sources [32–35]. In some systems the probe pulse is not even necessary as the pump both excites the response being investigated and engages the probing behaviour simultaneously. One example of this is in time-resolved Kelvin probe force microscopy (KPFM) experiments that measure the surface photovoltage of a sample as a function of time after a light source is pulsed. This was first implemented by Takihara et al. to measure the photovoltage dynamics of a sample at time scales faster than the KPFM feedback loop can track [36]. In this measurement mode, the tip–sample coupling is in an ‘always-on’ state and the time resolution is achieved by modulating the length of time the system is allowed to decay (i.e., the pulse-off time). The minimum time resolution is no longer limited by the detection electronics, but instead is theoretically limited only by the thermal noise of the cantilever [18]. This principle can be easily extended to ionic systems (such as those discussed previously) by simply replacing the pulsed light source with a pulsed voltage. In this case, the electric field engages the tip–sample coupling and simultaneously moves the mobile ions in the substrate, which leads to a changing tip–sample capacitance. Since the applied voltage controls the tip–sample coupling, turning the voltage off decouples the tip from changes occurring in the sample, thus the ionic transport is only probed during the pulse-on time, which can be directly controlled. Finding a relationship between the average frequency shift 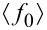 and the relaxation time constant of the sample τ* as a function of the pulse width *T* then allows for the sample transport dynamics to be extracted beyond the time resolution of the detection electronics. To relate the frequency shift of a cantilever to the tip–sample forces for FM-AFM, we turn to canonical perturbation theory using action-angle variables similar to the work done by Giessibl [37].

#### Derivation using canonical perturbation theory

Starting with the Hamiltonian for a harmonic oscillator and using a capacitive force perturbation Hamiltonian (Δ*H* = (1/2)*CV*^2^), we transform the momentum and position variables to action and angle variables: (*p*,*q*)→(α,β), where the first-order perturbation solution for the angle variable β_1_ (not to be confused with the earlier use of β as the exponential stretching factor) has the property:

[2]
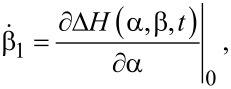


where the dot denotes the time derivative and the subscript 0 indicates that α and β are to be replaced with their unperturbed, constant values (α_0_,β_0_) after differentiation [38].

Writing *q* in terms of the action and angle variables explicitly:

[3]
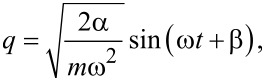


we see that 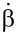 is the time derivative of the phase change due to the perturbing force. Taking the average therefore gives us the steady-state frequency shift 
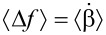
, which can easily be measured. Re-writing:

[4]
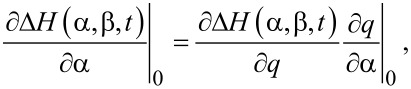


it can be easily shown that

[5]
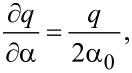


and 
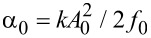
 where *A*_0_ is the oscillation amplitude, *k* is the spring constant, and *f*_0_ is the resonance frequency of the oscillator. In the simplest experiment where a time-varying voltage is applied between a conducting tip and sample, *C* has only an explicit dependence on *q* and *V* only an explicit dependence on *t*:

[6]
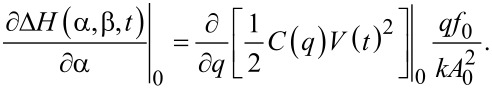


However, *C* does have an implicit time-dependence because the position *q* is not constant. The capacitance *C*(*q*) between a conducting sphere and conducting plane is approximated by [39]:

[7]
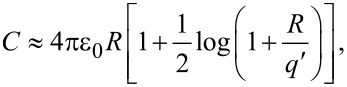


where *R* is the radius of the sphere and *q*’ is the sphere-plane (tip–sample) separation, which changes as the (tip) position, *q*, oscillates. In other words: 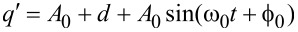, where 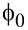 is the initial cantilever phase, ω_0_ is the free resonance frequency, *A*_0_ is the amplitude, and *d* is the closest tip–sample separation of the oscillating cantilever. Assuming this is an acceptable approximation for an AFM tip and conducting sample, the average frequency shift can be written as follows:

[8]
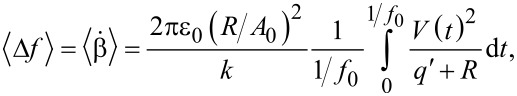


with the integral taken over one cycle (1/*f*_0_). Thus far we have only made an assumption regarding the functional form of the tip–sample capacitance. This relation (Equation 8) is thus valid for arbitrary oscillation amplitudes and timescales as long as the tip–sample interaction remains a small perturbation to the overall mechanical energy of the cantilever oscillation. This condition is fulfilled for a periodic voltage pulse with its frequency, *f_V_*, away from any of the mechanical resonances of the cantilever (*f_V_* *< f_i_*_+1_ and *f_V_* ¿ *f_i_* where *f_i_* is the *i*-th mechanical eigenfrequency of the cantilever). This is due to the large quality factor enhancement present on resonance, which would lead to a significant contribution to the total mechanical energy from even a small voltage (and thus field) applied near resonance, invalidating the perturbation approach to derive Equation 8.

#### Validation measurement

**Figure 3:**
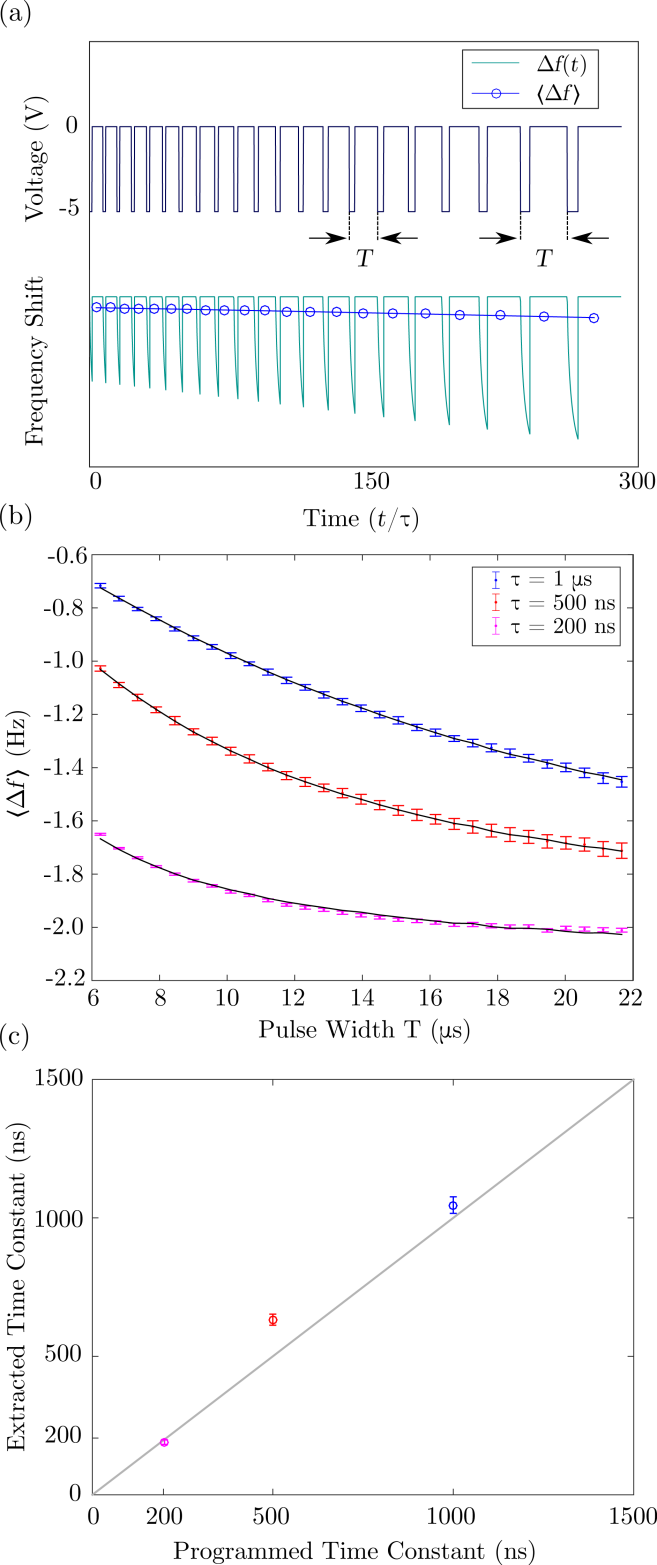
(a) Schematic illustration of the voltage-pulse averaging EFM technique: the top shows the applied voltage pulses with different pulse width (*T*), while the bottom shows the (simulated) instantaneous (Δ*f*) and average (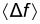) frequency shift due to an exponentially varying sample response. (b) Measured average frequency shift response as a function of the pulse width (*T*) for exponential voltage pulses with 1 μs (blue), 500 ns (red) and 200 ns (purple) time constants (τ), cantilever *f*_0_ = 16.7 kHz, 2 s averaging per measurement, 20 measurements per point. Error bars are the standard deviation of 20 measurements, and the black lines show fits to Equation 8. (c) Extracted time constant, (τ), as a function of the programmed time constant for the three measurements in (b); the solid gray line has a slope of unity to illustrate where points would lie for a perfect 1:1 relationship. Measured values: τ = 1.05 ± 0.03 μs, τ = 633 ± 20 ns, τ = 192 ± 3 ns. Note that a decay time of 190 ns is ca. 300-times faster than the cantilever oscillation period.

To demonstrate the time resolution of this technique a validation measurement was performed using a cantilever with a low resonance frequency (16.7 kHz) and a conducting tip over a gold sample. The tip was retracted a short distance (ca. 20 nm) with the *z*-feedback turned off and a train of exponential voltage pulses was applied, resulting in a change in the average frequency shift as a function of the width of the voltage pulse (*T*), illustrated in Figure 3a. The frequency shift was averaged over several seconds for each value of *T*, and *T* was then stepped as illustrated in Figure 3b. This full measurement was repeated 20 times with the *z*-feedback turned on and then back off between each measurement to minimize drift. Each pulse had the form *V*(*t*) = *V*_0_ + Δ*V*(1 − exp[−*t*/τ)] during the pulse-on period and *V*(*t*) = *V*_0_ during the pulse-off period with a duty cycle of 20%. To fit the data, the integral in Equation 8 was performed piecewise over the corresponding on and off time periods for one full cantilever oscillation: (0→*T*/5, *T*/5→*T*), (*T*→6*T*/5, 6*T*/5→2*T*), … , (…, (5*N* − 4)*T*/5→*NT*), where *NT* ≈ 1/*f*_0_ (note that *NT* ≠ 1/*f*_0_ because the pulse width cannot be an integer multiple of the oscillation period as discussed above). This integral has no closed-form solution and therefore must be computed numerically in order to fit the data. Since the phase between the applied pulses and the cantilever oscillation is arbitrary, the integral must be computed for many oscillation cycles (starting with an arbitrary phase) and then averaged to minimize the effect of the initial relative phase.

The results are shown in Figure 3 along with the fits to Equation 8 where we set: *V*(*t*) = *A* + *B*·exp(−*t*/τ), giving three fit parameters: *A*, *B*, and the time constant τ. The effect of the finite number of oscillation cycles appears in the fitted curves (black lines in Figure 3) as small deviations from a perfectly smooth function. This can be minimized by integrating over more cycles at the expense of increased computation time, which can be significant and has a negligible effect on the extracted fit parameters. The extracted time constants are plotted as a function of the programmed time constants in Figure 3c.

Extending this technique to ionic transport systems requires only the insertion of an explicit time dependence of the capacitance *C*(*t*) in place of the time-dependent voltage in Equation 8. The capacitance follows the time dependence of the system after a bias is applied, which is typically a stretched exponential as in Equation 1. Although this is in principle feasible, the main challenge is to perform the fitting. We attempted to fit the data in Figure 3 to a stretched exponential with an additional parameter, β, which should result in an extracted value of β = 1 since this is a ‘pure’ exponential decay. The fitting was very problematic due to the dependence of the fit results on the chosen initial conditions. This is a general challenge when using functions with numerous fit parameters, in accordance with the famous quote about fitting an elephant by John Von Neumann [40].

#### Assumptions and limitations

As shown by this validation measurement, this technique can be used to measure transport processes occurring faster than the period of the cantilever. Fundamentally, the time resolution should only be limited by the minimum electrical pulse width that can be reliably applied to the sample (which is likely much larger than the theoretical limit [18]). The only assumption used that may not be true for all cases is the functional form of the tip–sample capacitance (Equation 7). To test the accuracy of this assumption we investigated both a conducting sample and a thick dielectric sample (200 μm thick sapphire, ε_r_ = 11.3) by measuring the frequency shift as a function of the distance with a constant applied bias. The force was then extracted from the frequency shift using the Sader–Jarvis method [41] while taking care to ensure that the inversion procedure is mathematically well-posed for this particular experiment [42–43].

**Figure 4:**
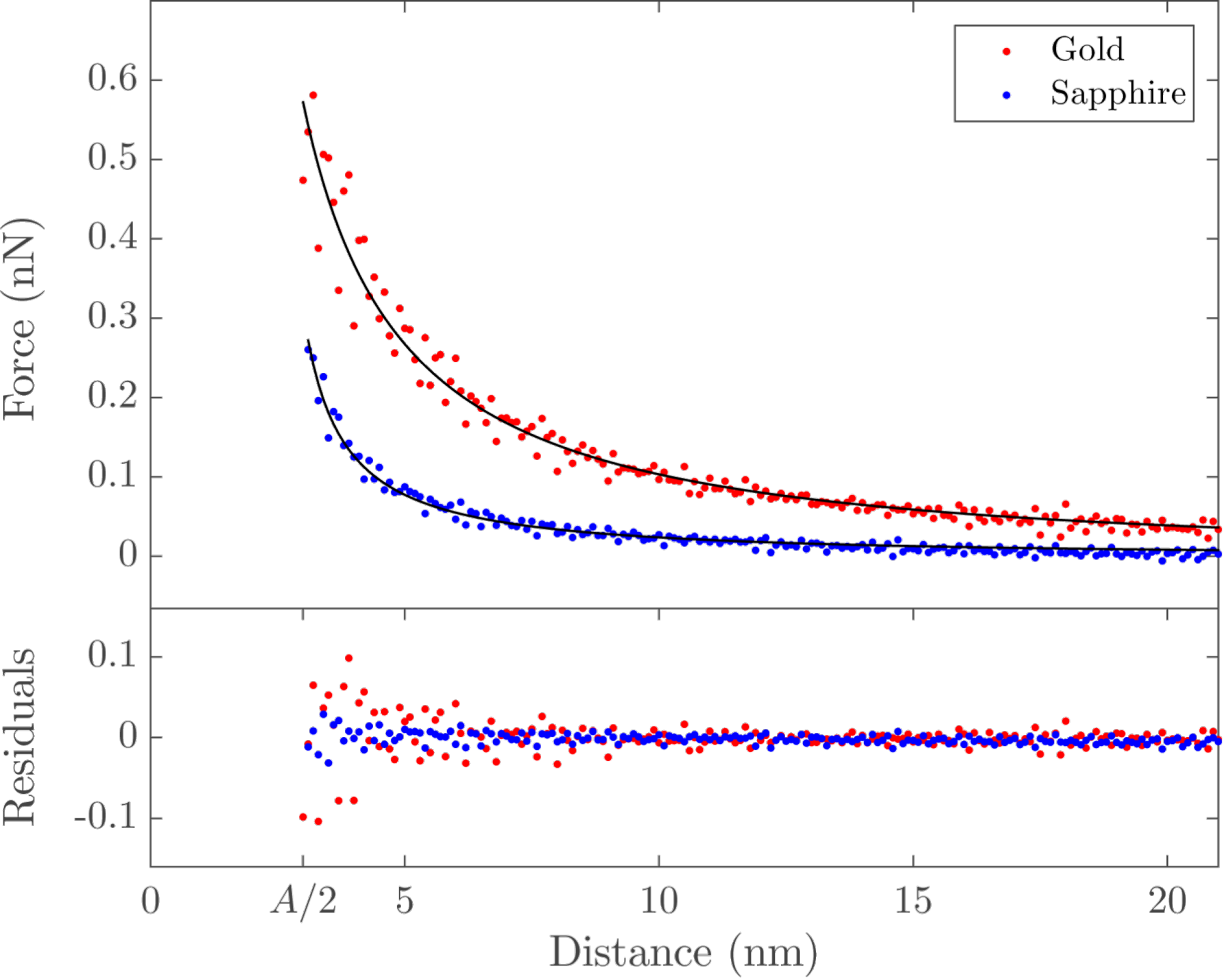
Measured tip–sample force as a function of the distance for a gold-coated tip over a grounded gold substrate (red) and a grounded 200 μm thick sapphire substrate (blue) with 4 V applied to the tip. Cantilever parameters: *f*_0_ = 295.621 kHz, *k* = 27 N/m, *A* = 6 nm (Mikromasch NSC15/CR-AU).

The results are shown in Figure 4 where the black lines are fits to the approximate force between a conducting sphere held at a constant potential and a conducting plane:

[9]
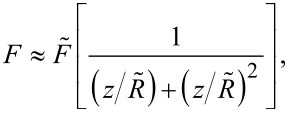


where 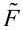 and 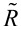 are fit parameters. Theoretically, 
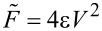
, where ε is the permittivity and *V* is the applied voltage, and 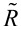 is the effective tip radius. The oscillation amplitude for both experiments was 6 nm, thus the *x*-axis is the average tip–sample separation and the zero-point was chosen as the point when the oscillation stopped due to contact with the sample. The resulting values for 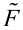 were 0.6 nN for gold and 38.5 nN for sapphire and the effective tip radii obtained were 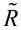 = 4.3 nm and 0.23 nm for gold and sapphire, respectively. The 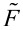 value obtained for gold is very close to the theoretical value of 0.53 nN, but the value for sapphire is off by approximately a factor of 5, while the tip radii are significantly smaller than the true tip radius (ca. 30 nm). These results are not surprising as there are many potential sources of error that can affect the absolute value of the force including the calibration of the cantilever spring constant, background forces from the conical probe and the cantilever itself, and uncertainty in the zero-point for both the tip–sample separation and the force itself, which are typically chosen arbitrarily [44–49]. Note that this experiment aims only to demonstrate the validity of the functional form of the relationship and not as a quantitative measurement of these parameters. Better approximations than the simple sphere–plane one used here have been developed, but would introduce significant challenges in computing the integral in Equation 8. Using this simple approximation for both gold and sapphire substrates, the residuals are normally distributed to within experimental uncertainty (i.e., the χ^2^ goodness of fit test performed on the residuals does not reject the null hypothesis to 0.05 significance [50]). This demonstrates that the functional form of this approximation is valid with this particular probe type on samples of two extremes (a smooth conductor and a thick dielectric material). However, it is not necessarily valid in all cases and should therefore be verified through spectroscopy measurements such as this on a case-by-case basis.

Sapphire was chosen for its high dielectric constant (ε_r_ *>* 10), which is similar to those found in many solid ionic conductors such as LiFePO_4_ and LiCoO_2_, and for its low electronic conductivity and lack of mobile ions. This experiment is therefore a reliable validation of the *z*-dependence of the tip–sample capacitance expected for actual ionic transport measurements on relevant samples.

### Fast free time-resolved EFM

#### Motivation

**Figure 5:**
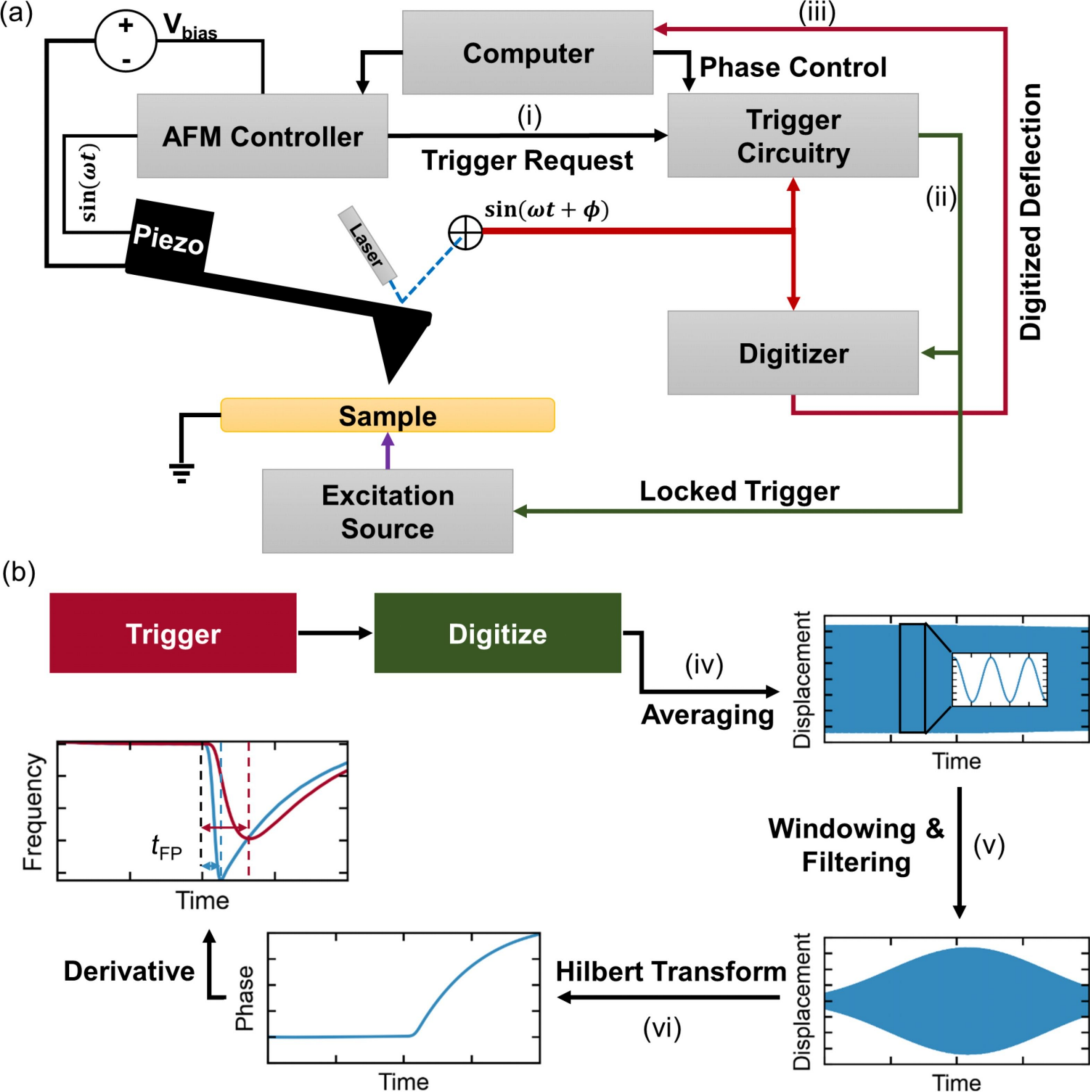
“(a) Schematic of the experimental setup: (i) Computer requests a trigger at a defined phase of the oscillation, (ii) trigger circuitry triggers the digitizer and the excitation source accordingly, and (iii) the digitizer transfers the acquired signals to computer. (b) Flow diagram of the data analysis procedure after the acquisition: (iv) signals are averaged together, (v) windowed and filtered, and (vi) demodulated via Hilbert transform to find the instantaneous frequency of the cantilever (instantaneous frequency curves for excitations with time constants of 25 ns and 100 μs are shown).” Reproduced with permission from [51], copyright 2016 AIP Publishing.

It is clear that there are challenges in using time-averaged AFM signals to extract fast sample dynamics, namely a priori knowledge or assumptions of the specific temporal functional form of the dynamics. Some techniques have sought to avoid this by directly capturing the deflection signal using high-speed data acquisition systems and performing offline analysis to reconstruct the sample response. One such technique is fast free time-resolved electrostatic force microscopy (FF-trEFM), first proposed by Giridharagopal and co-workers [22]. FF-trEFM captures the full dynamics of an oscillating cantilever when an interaction force between the tip and sample is turned on. An overview of this technique is shown in Figure 5 (reproduced from [51]).

#### Description and implementation

To implement FF-trEFM requires the addition of a high-speed data acquisition system to a standard AFM, which is not overly expensive or onerous. Acquiring the raw deflection signal in the time-domain precludes the necessity for expensive detection electronics that are commonly used to acquire and demodulate the oscillation of the cantilever. The only limitation on standard AFM systems are the photodetectors, which typically have bandwidths of 1–2 MHz, although faster photodiodes are available. The raw signal can then be filtered and postprocessed using a Hilbert transform to extract the analytical signal and what is known as the ‘instantaneous frequency’ (the time derivative of the instantaneous phase). Examining the extracted instantaneous frequency after applying a voltage with an exponential rise time τ (shown in Figure 5b for simulated data), it is clear that the response shows observable differences as a function of τ. Since the cantilever is continuously driven throughout the experiment, the instantaneous frequency shows a fast transient response to the applied pulse, followed by a slow relaxation towards a new steady-state value. This leads to a clear initial peak in the frequency shift, which is defined as the ‘time to first frequency shift peak’ (*t*_FP_) by Giridharagopal and co-workers [22]. The authors demonstrated that simulated results (both numerical simulations of a damped-driven harmonic oscillator and finite element simulations) and their experimental results show excellent agreement given the same parameters and subject to the same postprocessing (windowing, filtering, and analytical signal extraction).

It is instructive to note that the extracted instantaneous frequency contains a time delay introduced by the bandpass filter used in the processing to smooth the response, which cannot be completely corrected for. This leads to the attenuation of high-frequency components, especially for decay times faster than the oscillation period. Because of this attenuation, the extracted signal is a representation of the true ‘instantaneous frequency’, leading to difficulties in determining the full functional form of the time-dependent tip–sample interaction.

#### Application to ionic transport measurements

To accurately quantify ionic transport requires the capability to fully resolve the functional form of a stretched exponential response to extract the two main parameters of interest, τ and β. To test the suitability of FF-trEFM for these measurements, we performed numerical simulations (of a damped driven harmonic oscillator, see Supporting Information File 1 for MATLAB code) similar to those performed by Karatay and co-workers [51], using instead a resonance frequency varying in time as a stretched exponential and a stretched exponential electrostatic force term:

[10]
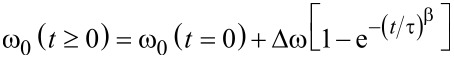


and

[11]
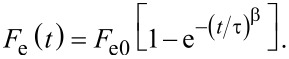


**Figure 6:**
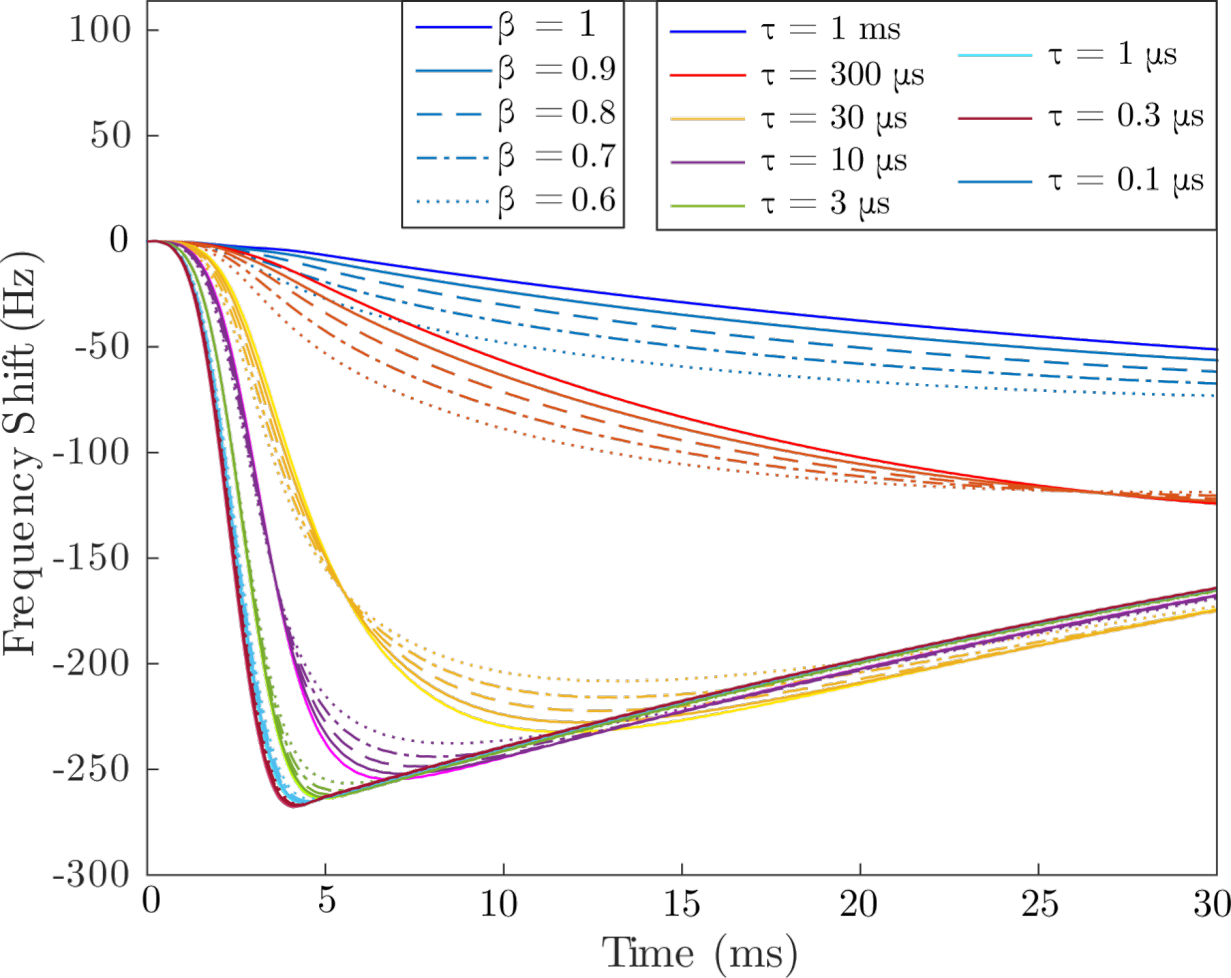
Extracted FF-trEFM signals from numerical simulations of tip–sample interactions with a stretched-exponential time response (Equation 1) with various relaxation time constants, τ, and stretching factors, β.

The parameters employed were ω_0_ = 2π × 277 kHz, Δω = 2π × 277 Hz, and *F*_e0_ = 3 nN, similar to those used in [22]. The results after windowing, filtering, and performing the Hilbert transform are shown in Figure 6. The colours denote different time constants and the β values are shown by different line styles. For slower time constants (τ ≥ 10 μs) the different values of β are visually distinct; however, at much smaller timescales these distinctions are no longer visible, making ionic transport measurements using FF-trEFM challenging and possibly no more advantageous than direct time-domain EFM.

#### Demonstration of spatial resolution

**Figure 7:**
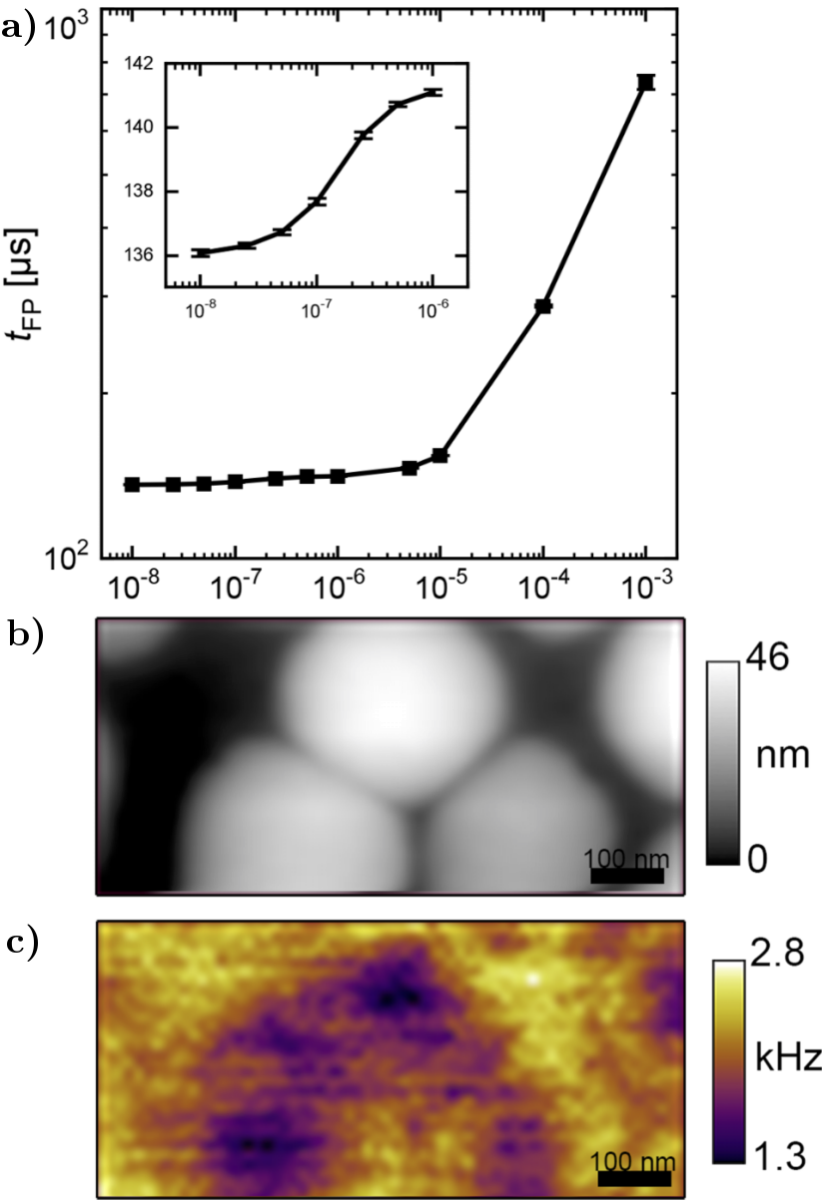
“(a) Calibration curve for a range of characteristic times of exponential decay (τ) (inset shows a zoom-in for shorter times). (b) Topography and (c) inverse τ_FP_ (τ_FP_^−1^) images of a 1:4 ratio MDMO-PPV:PCBM thin film photovoltaic device cast from toluene. Excitation wavelength is 488 nm and intensity at the tip is *~*290 W/cm^2^. Data are acquired at 10 nm lift height, 10 V bias between the cantilever and the sample, with 60 averages per pixel.” Adapted with permission from [51], copyright 2016 AIP Publishing.

In more recent work by Karatay and co-workers, they analyzed how a variety of factors including noise and the phase difference between the cantilever oscillation and the applied pulses affect the achievable time resolution [51]. They presented guidelines for implementation of their technique, in particular the use of photothermal excitation to reduce other sources of mechanical noise. To study the relationship between the system dynamics and the measured *t*_FP_ response they mapped *t*_FP_ as a function of true exponential time constant τ to generate a calibration curve (Figure 7a). They observed statistically significant differences in the measured signal in differences in τ down to 10 ns, which they designated as the minium attainable time resolution. The authors then utilized *t*_FP_ to study differences in local charging times of an organic photovoltaic thin film (MDMO-PPV:PCBM), shown in Figure 7, and demonstrated the ability of the technique to spatially resolve heterogeneities. Due to the difficulty in quantitatively extracting τ from the measured τ_FP_, spatially resolved measurements are limited to relative charging rates presented as spatial mapping of τ_FP_. These results can still provide useful insight into sample dynamics (in this case the quantum efficiency of the photovoltaic material) even though direct quantitative measurements of decay time constants may not always be possible.

### Phase-kick EFM

#### Background and implementation

**Figure 8:**
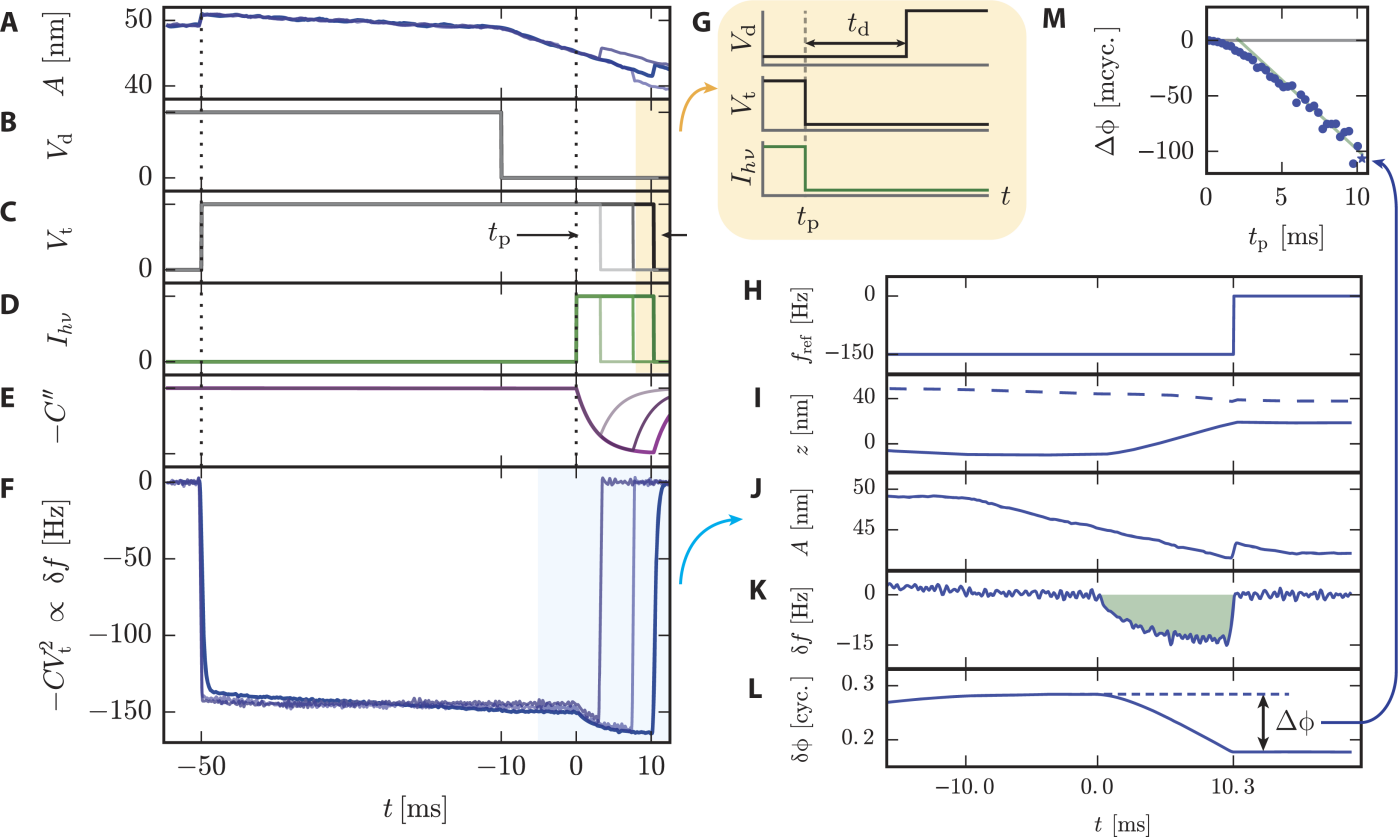
“For three representative pulse times, we plot (A) cantilever amplitude; (B) cantilever drive voltage, turned off at *t* = −10 ms; (C) tip voltage, with the pulse time *t*_p_ indicated; (D) sample illumination intensity, turned on at *t* = 0; (E) sample capacitance; and (F) cantilever frequency shift. (G) Timing of applied voltages and light pulses. The voltage and light turn off simultaneously at *t* = *t*_p_. After a delay *t*_d_ (typically 5 to 15 ms), the cantilever drive voltage is turned back on. Next, we illustrate how the phase shift 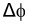 is calculated using the *t*_p_ = 10.3 ms data. We process the cantilever displacement data using a software lock-in amplifier. (H) The software lock-in amplifier reference frequency changes at *t* = *t*_p_. The software lock-in amplifier outputs (I) the in-phase (solid) and out-of-phase (dashed) components of the cantilever displacement; (J) cantilever amplitude; (K) frequency shift; and (L) phase shift. The total phaseshift 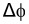 is equal to the highlighted area under the cantilever frequency shift curve. (M) The voltage- and light-induced phase shift 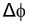 is measured as a function of the pulse time *t*_p_. We show only every other data point for clarity. The *t*_p_ = 10.3 ms data point is denoted with a star. Experimental parameters: PFB:F8BT on indium tin oxide (ITO) film, *h* = 250 nm, *V*_t_ = 10 V, *I_hν_* = 0.3 kW m^−2^, delay time between pulses = 1.5 s.” The cantilever used had a resonance frequency of 62 kHz. Reproduced with permission from [23].

Another technique recently developed by Dwyer and co-workers, referred to as “phase-kick” EFM (pk-EFM), uses an indirect measurement of the cumulative change of a cantilever parameter (phase or amplitude) in order to reconstruct a time-varying signal [23]. One implementation of pk-EFM utilizes a carefully timed voltage pulse applied between tip and sample that controls the tip–sample coupling while a light pulse is also applied, as illustrated in Figure 8 (reproduced from [23]). Initially, the cantilever is driven on resonance at a steady-state amplitude and a voltage is applied. The voltage engages the tip–sample coupling and leads to an initial frequency shift, which can be seen at *t* = −50 ms in Figure 8F. A short time later the drive is turned off so that the cantilever is freely oscillating; practically, this removes the drive signal as a source of noise in the experiment. At *t* = 0 a light pulse is then applied and the capacitance varies temporally as the sample charges due to the photoexcitation. By then abruptly turning off the tip–sample coupling (by setting the voltage back to 0) the total photocapacitance change measured by the cantilever can be controlled. The applied voltage therefore acts as a gate that controls the cumulative sample response that is captured in the cumulative change in the cantilever oscillation. The total phase shift 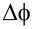 from the time the light pulse is applied (*t* = 0) to the time when the voltage is returned to 0 (*t* = *t*_p_) is then proportional to the integrated photocapacitance since the voltage is held constant over this time:

[12]
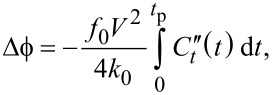


where 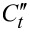 is the is the second derivative of the tip–sample capacitance with respect to vertical separation, *f*_0_ is the resonance frequency, and *k*_0_ is the spring constant. This result is derived from the relationship between the frequency shift and the capacitive force between the tip and sample:

[13]
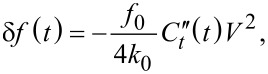


which is a valid approximation in the limiting case of small oscillation amplitudes [37]. More specifically, this approximation is only valid if the force gradient is constant over one full oscillation of the tip [52]. This can be achieved under typical experimental conditions (1–5 nm oscillation amplitude) by simply performing the measurement with a larger tip–sample separation, but this comes at the cost of degraded spatial resolution. Achieving smaller oscillation amplitudes (much smaller than 1 nm) is possible using more sensitive detection methods (interferometry, for example [53]) and cleaner excitation schemes such as photothermal excitation [54]. Using probes of higher stiffness, however, is not expected to be advantageous due to the inverse relationship between the measured phase shift and cantilever spring constant.

#### Validation measurement

To demonstrate the sub-cycle time resolution of this technique, Dwyer et al. used the Magnus expansion in order to solve the system of linear differential equations describing the cantilever motion [23]. Modelling the photocapacitance as a single exponential with a risetime of τ,


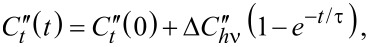


resulted in two expressions relating the cumulative amplitude (Δ*A*) and phase shifts (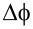) to the time constant:

[14]
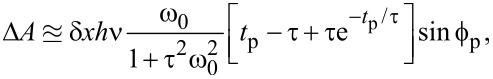


**Figure 9:**
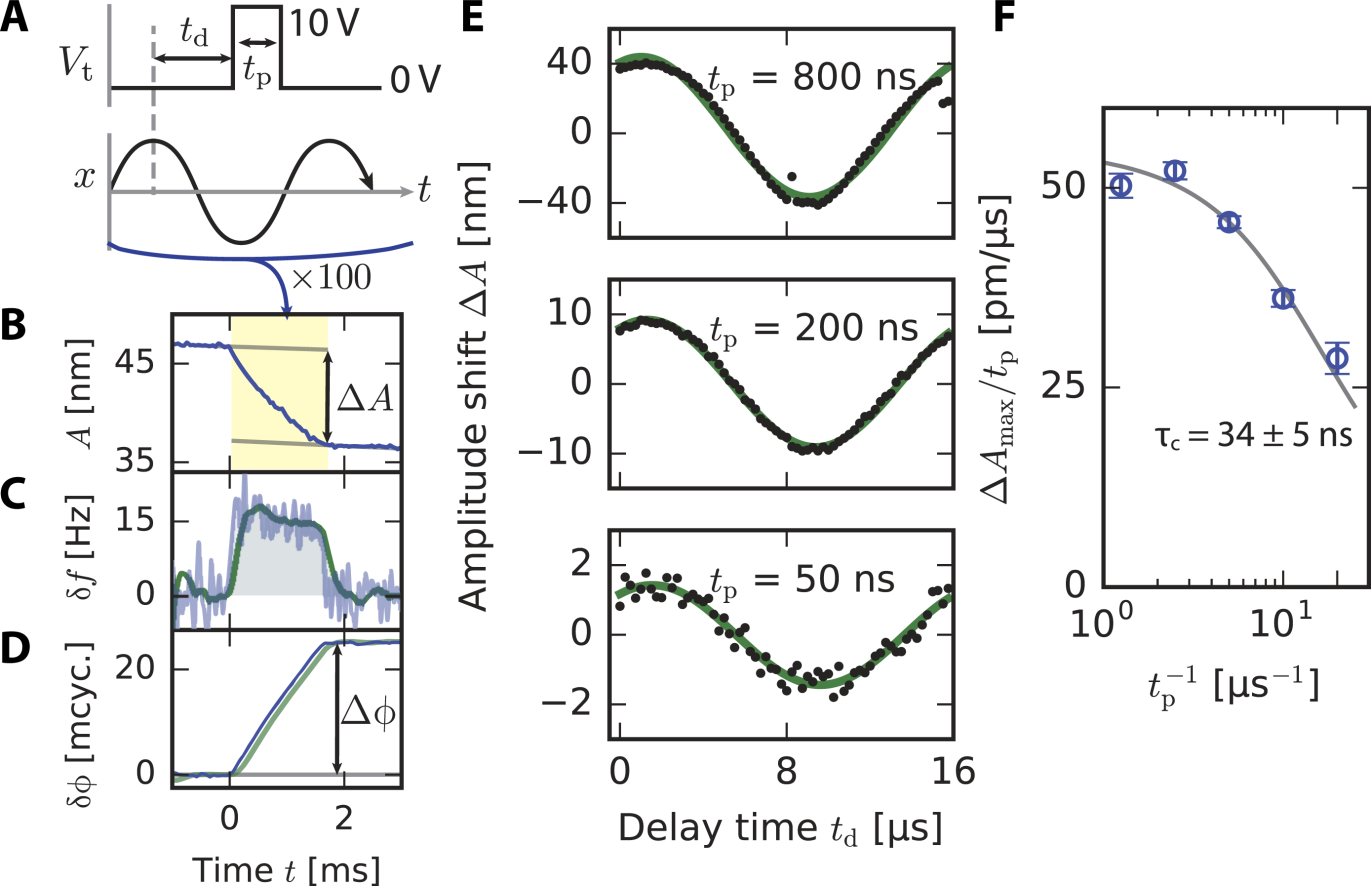
“Experiments and simulations demonstrating subcycle time resolution in pk-EFM. (A) Subcycle voltage-pulse control experiment (PFB:F8BT on ITO, *h* = 250 nm). A voltage pulse of length *t*_p_ is applied to the cantilever tip (top) at a delay of *t*_d_ relative to the cantilever oscillation (middle) for 100 consecutive cantilever oscillations. (B) The pulses shift the cantilever amplitude by Δ*A*.(C) Measured frequency shift and (D) phase shift, demodulated with a 3-dB bandwidth of 4.8 kHz (blue) and 1.5 kHz (green). (E) The amplitude shift Δ*A* versus delay time *t*_d_ for three representative pulse lengths. (F) The normalized response Δ*A*_max_/*t*_p_ obtained by fitting data in (E) shows the cantilever wiring attenuating the response at short pulse times. The gray line is a fit to a single-exponential cantilever charging transient.” Adapted with permission from [23].

[15]
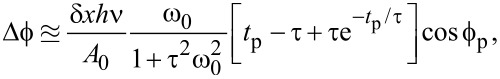


where 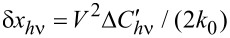 (δ*x_hν_* is the dc deflection due to the photocapacitive force). This result is valid for very short times after the voltage is abruptly returned to 0. It is especially interesting as it relates the change in amplitude and phase with the phase of the cantilever when the voltage is turned off, 
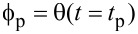
. By tuning *t*_p_, a phase shift, an amplitude change, or a combination of both can be induced. The technique is fully illustrated in Figure 9 where the amplitude data in Figure 9E (Δ*A*) was obtained by voltage pulses alone. The voltage pulses resulted in charging/discharging of the sample (PFB:F8BT on ITO), which was also modelled as a single exponential in time: *V_t_*(*t*) = *V*[1 − exp(−*t*/τ_c_)]. This allowed for the amplitude to be written as a function of *t*_p_ and τ_c_ where it again displayed a sinusoidal dependence on 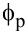. Note that *t*_p_ in Figure 9 refers to the width of the voltage pulse, whereas *t*_p_ used in the derivation was the time at which the voltage was returned to zero. Relabelling the width of the voltage pulses as 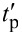, this yields 
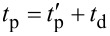
; in other words the *t*_p_ in Equation 14 and Equation 15 can be tuned by changing the delay time *t*_d_. This is shown in Figure 9E where the sinusoidal behaviour of Δ*A* as a function of *t*_d_ is clear. Figure 9F shows the maximum amplitude change Δ*A*_max_ as a function of the inverse pulse width 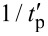. An exponentially decreasing amplitude change at smaller pulse widths is clearly visible, which the authors explain by the charge being unable to get in and out of the sample on these fast timescales. The extracted time constant for the charging time, τ_c_, was 34 ± 5 ns.

#### Application to ionic transport measurements

From this voltage-pulse measurement it is clear that this technique can easily be extended to measure ionic transport. In fact, it may even be best suited to this application as it requires only a precisely timed voltage pulse instead of phase-locked voltage and light pulses. This technique operates in much the same way as the voltage-pulse averaging method previously described: A parameter of the cantilever oscillation (be it phase, frequency shift, or amplitude) is averaged over a long time period while a coherent (i.e., phase-locked with respect to cantilever oscillation) repeating signal is applied over a much smaller time period that induces a change in the measured parameter. By changing the length of time that the ‘fast’ signal is allowed to interact with the cantilever it can then be reconstructed by relating the slowly varying parameter to the fast dynamics. Although similar to pump–probe style measurements, these techniques are unique in that they operate by changing the cumulative interaction time between the probe and the sample instead of simply capturing ‘snapshots’ of the evolution of the sample dynamics as a function of time. This allows for sample dynamics that are driven only while the tip–sample coupling is engaged to be measured, such as ionic transport, for example. Directly applying this technique to measure ionic transport, however, would require the addition of a stretching factor, β, into the exponential as previously discussed. The main complication in this case is performing the time-integral over the capacitive gradient, which was assumed to be a simple exponential in Equation 14 and Equation 15. The integral will not have a closed-form solution, which would require either a series expansion approximation or a numerical approximation in order to extract useful information from the data. This will likely result in the same challenge as we encountered with the voltage-pulse averaging technique where the least-squares fitting has many local minima for the fit parameters resulting in a strong dependence of the fit results on the initial conditions. Nonetheless, this technique is promising in terms of achieving better time-resolution in EFM-based measurements.

### Intermodulation spectroscopy

#### Background and implementation

**Figure 10:**
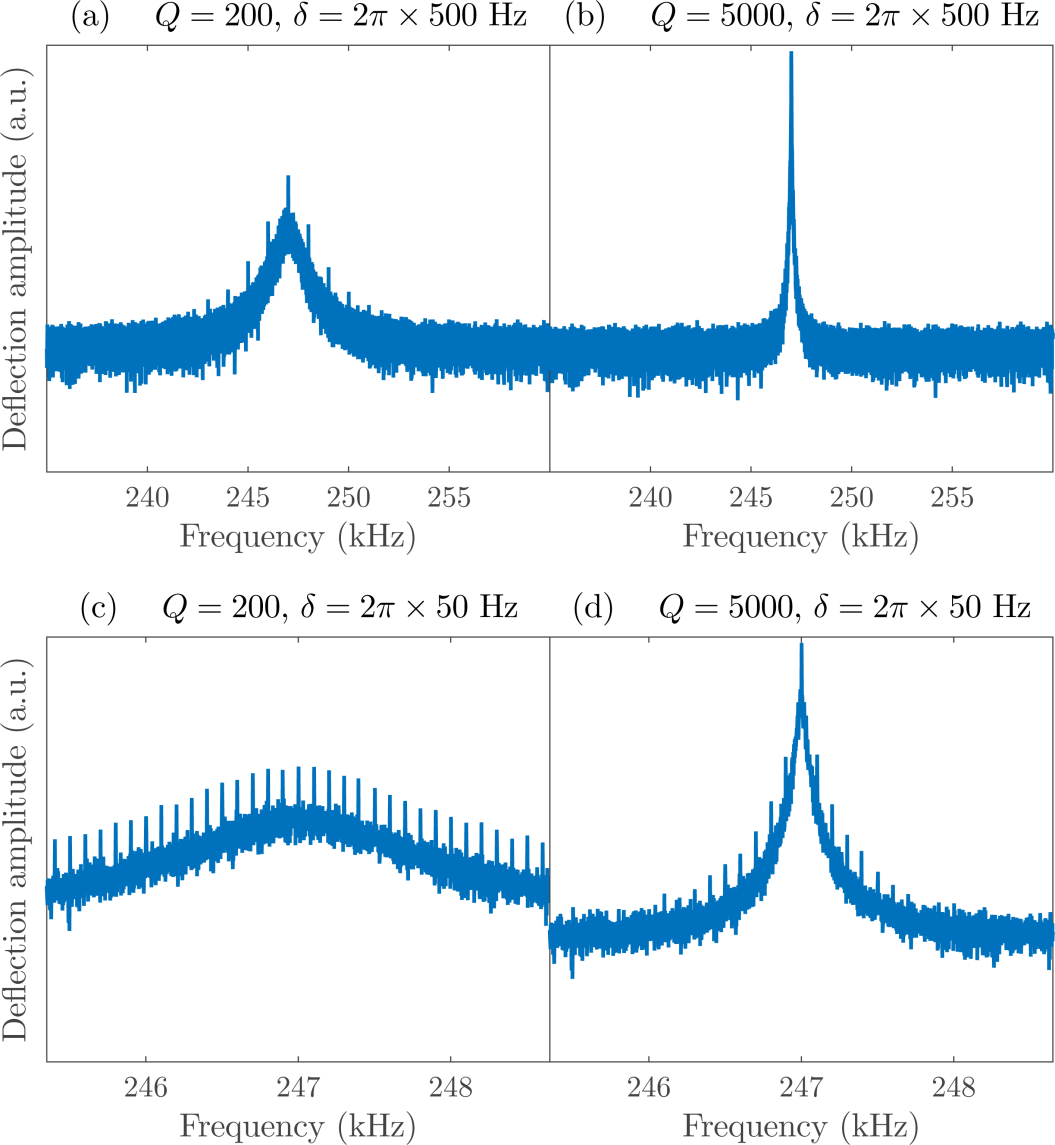
Synthetic data of cantilever deflection spectrum around the fundamental resonance frequency, ω_0_ =2π × 247 kHz, with: (a) δ = 2π × 500 Hz and a quality factor typical for ambient measurements, *Q* = 200; (b) δ = 2π × 500Hz and *Q* = 5000, typical of vacuum measurements; (c) δ = 2π × 50 Hz and *Q* = 200; and (d) δ = 2π × 50 Hz and *Q* = 5000.

Significant progress has clearly been made in measuring electrostatic force microscopy signals in the time domain. The main challenges of the techniques discussed thus far have been the detection methods (specifically, bandwidth limitations) and various assumptions and approximations that have been made, which limit the useful parameter space of some experiments. Looking instead in the frequency domain, one very recent method of extracting fast sample dynamics appears to be a promising alternative to many of these challenges. Intermodulation spectroscopy, developed by Borgani and Haviland [20], utilizes the spectral response of a cantilever near resonance due to an applied pulse train (optical or electrical) in order to probe sample dynamics. This technique exploits the non-linear tip–sample interaction due to the applied pulse train that results in a spectrum of peaks at various sum and difference frequencies, illustrated in Figure 10. Each of these frequency components (referred to as intermodulation products, or IMPs) contains information about the interaction, which can be extracted by looking at the Fourier series expansion of the tip–sample interaction. Since the interaction is purely capacitive, the force is given by


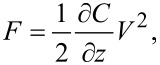


which contains two separate variables each with their own periodicity: the capacitive gradient ∂*C*/∂*z*, and the voltage *V*. Since *V* is controlled by the applied excitation, it is periodic in ω_E_, the repetition rate of the applied pulses. The Fourier series for *V*^2^(*t*) is therefore given by:

[16]
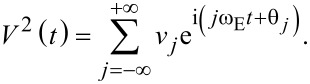


In the case where the sample capacitance remains constant (in a conducting sample, for example), the capacitance gradient has the same periodicity as the cantilever oscillation since the cantilever sweeps through the gradient as it oscillates. The Fourier series expansion for


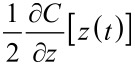


can then be written as

[17]
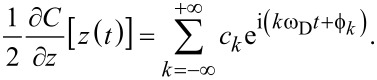


The authors proposed three distinct excitation schemes based on the frequency of the applied pulses, ω_E_, relative to the mechanical drive frequency ω_D_: resonant excitation, where ω_E_ = ω_D_ + δ, with 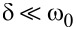; sub-resonant excitation, where 
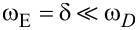
; and super-resonant excitation, where ω_E_ = 2ω_D_ + δ. For each excitation scheme they showed the Fourier coefficients for the first six IMPs, which have the form


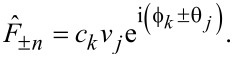


For resonant excitation, they determined that taking the ratio and product of certain pairs (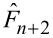 and 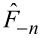) yields quantities that depend only on the electrical response, which completely eliminates the dependence on the capacitance gradient. Thus, to extract information about the system using these quantities does not require any assumptions about the functional form of the capacitance gradient. The only (major, and possibly limiting) assumption is that the sample is metallic (see discussion below). The authors also derived similar ratios for both the sub-resonant and super-resonant schemes, allowing them to directly compare the time resolution and signal-to-noise ratio of each.

#### Validation measurement

**Figure 11:**
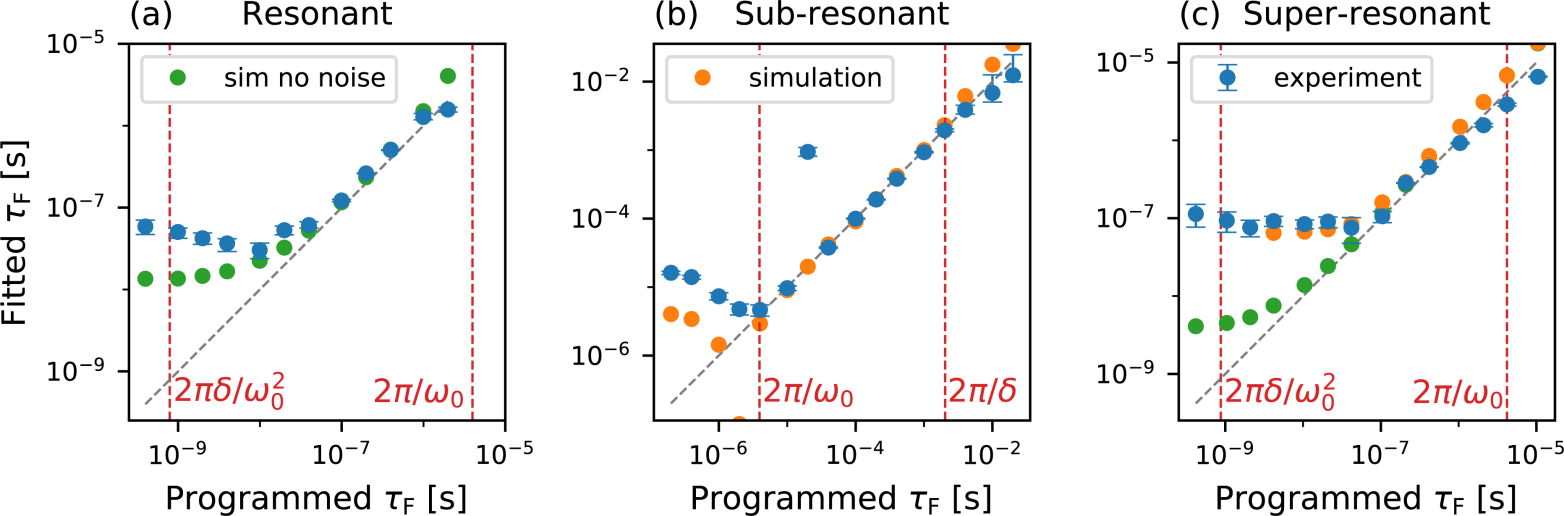
“Results from simulations and from the experimental validation for the proposed excitation schemes: (a) resonant, (b) sub-resonant and (c) super-resonant. The cantilever has a resonance frequency of about 2π × 250 kHz, a value of δ = 2π × 500 Hz is used in the sub-resonant scheme and δ = 2π × 50 Hz in the resonant and super-resonant schemes. The fittted values τ_F_ are plotted versus the value programmed in the simulation and in the MLA. The gray dashed lines have slope unity and indicate where a perfect data point would be. For the experimental data, a series of 256 measurements is performed at each value of programmed τ_F_: the blue dots indicate the median of the reconstructed values, and the error bars indicate the inter-quartile range. The vertical red dashed lines mark the time resolution calculated in Sec. IV.” of [20]. “(a) in the resonant scheme, both simulations without noise (green dots) and experiments fail to reach the predicted time-resolution, due to the violation of Eq. (7). (b) in the sub-resonant scheme, simulations with detector and force noise (orange dots) and experiments show the predicted time resolution. (c) in the super-resonant scheme, simulations without noise approach the predicted time resolution, while experiments are limited to about 50 ns. Simulations with detector and force noise reproduce the experimental data.” Reproduced with permission from [20], copyright 2019 AIP Publishing.

As a validation measurement, they applied electrical pulses with known exponential charging times between a conducting tip and sample and extracted the rise and fall time constants, τ_r_ and τ_f_, using an analytical model for *V*^2^(*t*). This allows for a high-fidelity reconstruction of the true signal using only a few Fourier coefficients. Using the resonant excitation scheme, they accurately extracted the time constants down to ca. 20 ns, approaching the theoretical limit they derived for the technique. Their results are shown in Figure 11 for each of the three excitation schemes. Both the resonant and super-resonant schemes allowed signals more than an order of magnitude faster than the oscillation period of the cantilever to be extracted.

They report that mapping of system dynamics can be done even at standard imaging speeds due to the simultaneous acquisition at multiple frequencies using a multi-frequency lock-in amplifier. This is a drastic improvement over many other time-resolved EFM variations that require lengthy averaging times, which makes spatial mapping difficult and time consuming.

#### Challenges and application to ionic transport measurements

Thus far, measurements using this technique have only been performed under ambient conditions and on a conducting sample with known voltage pulses. One foreseeable challenge will be in performing measurements under vacuum conditions, which is typically beneficial due to the large increase in quality factor that leads to a greater force sensitivity. However, for intermodulation spectroscopy measurements, this will lead to a smaller frequency window in which quality factor enhancement will be available to boost the relative amplitudes of the IMPs. This is illustrated in Figure 10 where panel (a) shows synthetic data for a standard cantilever in ambient (*Q* = 200) with several IMPs clearly visible above the noise. Figure 10b shows the exact same simulated experiment with a much higher quality factor (*Q* = 5000), which is typical for vacuum applications. In both cases δ = 2π × 500 Hz. Reducing δ to 50 Hz, as was done for the resonant and super-resonant experiments, yields many more IMPs above the noise level as shown in Figure 10c. Simultaneously demodulating several of these components allows for accurate reconstruction of the time-varying voltage. However, in the case of high *Q*, we see that many of the peaks are now well below the noise level due to the size of the resonance peak compared to the IMP spacing (δ). The signal-to-noise ratio is significantly lower in this case, requiring a much smaller value of δ and, in turn, longer averaging times. Note that since the time resolution is proportional to δ (Equations 15 and 16 in [20]), using a smaller δ values actually results in better time resolution, at the expense of longer measurement times.

Another complication that may be encountered will be observed when performing measurements on samples with non-static capacitance gradients. For the analysis performed in this case the capacitance was assumed to have only the periodicity of the cantilever oscillation, ω_D_, which is, of course, valid because the sample is a conductor. This will not be the case when the capacitance gradient has an explicit time-dependence, as with many photovoltaic and ionic conductors. In these samples, the tip–sample capacitance gradient will evolve with time after the application of the pulse (be it optical or electronic) and will therefore have a frequency component matching that of the applied pulses, ω*_E_*. This is due to sample dynamics such as photoexcitation or ionic transport [12,23,55]. This may make extracting the time response of the sample much more difficult if the capacitive Fourier coefficients cannot be eliminated by taking ratios of certain components. There may be methods of minimizing this effect, especially in the case of optical pulses for measuring time-resolved photocapacitance similar to the pk-EFM method discussed previously [23]. In this implementation, Dwyer et al. applied a large bias between the tip and sample to engage the coupling, having the fortunate side-effect of rendering the measurement insensitive to small variations in surface potential as the sample charges. This results in a response that is only sensitive to the time-varying capacitance, simplifying the analysis significantly. Similar techniques may be required to extract information from samples where large time-dependent changes in capacitance and surface potential are expected.

## Conclusion

We have reviewed several established techniques that achieve time resolution using EFM and examined their assumptions, limitations, and potential applications. Direct time-domain EFM is the most straightforward to implement, but is limited to measuring on timescales much slower than the cantilever oscillation. The new technique we have demonstrated – voltage-pulse averaging EFM – allows for time resolution much faster than the cantilever oscillation period, but requires a priori knowledge of the time-evolution of the signal and the functional form of the tip–sample capacitance. FF-trEFM, which uses post-processing to extract the instantaneous frequency of the cantilever, allows for rapid data acquisition while scanning and high spatial resolution, but suffers from a nonlinear variation of the measured signal with the time constant of the sample response for fast responses. Phase-kick EFM provides a pathway to extract sample dynamics indirectly by observing cumulative changes in the cantilever oscillation, but relies on the assumption that the oscillation amplitude is small with regards to the capacitance gradient, which can be violated for large amplitudes and/or small tip–sample separations. Finally, we have looked in detail at intermodulation spectroscopy, which exploits the non-linear signal mixing of the cantilever oscillation and an applied pulse train by recording the various frequency components corresponding to specific Fourier coefficients. This technique may encounter difficulties in extracting information for measurements where the tip–sample capacitance also changes as a result of the applied pulse train.

Despite many of these assumptions and potential limitations, all of these techniques represent great strides in the advancement of time-resolution in EFM. With the need for measurements of faster and faster dynamics with higher spatial resolution, the role of time-resolved EFM as a key tool is more significant than ever.

## Supporting Information

File 1 MATLAB code to simulate FF-trEFM measurements with a stretched exponential response.
